# Influence of Elbow Angle on Erosion-Corrosion of 1018 Steel for Gas–Liquid–Solid Three Phase Flow

**DOI:** 10.3390/ma15103721

**Published:** 2022-05-23

**Authors:** Rehan Khan, Hamdan H. Ya, Imran Shah, Usama Muhammad Niazi, Bilal Anjum Ahmed, Muhammad Irfan, Adam Glowacz, Zbigniew Pilch, Frantisek Brumercik, Mohammad Azeem, Mohammad Azad Alam, Tauseef Ahmed

**Affiliations:** 1 Department of Mechanical Engineering, College of Electrical and Mechanical Engineering, National University of Sciences and Technology, Islamabad 44000, Pakistan; bilal.anjum@ceme.nust.edu.pk; 2 Mechanical Engineering Department, Universiti Teknologi PETRONAS, Seri Iskandar 31750, Malaysia; hamdan.ya@utp.edu.my (H.H.Y.); mohammad_18000380@utp.edu.my (M.A.); mohammad_18000664@utp.edu.my (M.A.A.); tauseef_17007229@utp.edu.my (T.A.); 3 Department of Aerospace Engineering, College of Aeronautical Engineering, National University of Sciences and Technology, Risalpur 24080, Pakistan; ishah@cae.nust.edu.pk; 4 Department of Mechanical Engineering Technology, National Skills University, Islamabad 44000, Pakistan; ukniaxi@gmail.com; 5 Electrical Engineering Department, College of Engineering, Najran University, Najran 61441, Saudi Arabia; miditta@nu.edu.sa; 6 Department of Automatic Control and Robotics, Faculty of Electrical Engineering, Automatics, Computer Science and Biomedical Engineering, AGH University of Science and Technology, Al. A. Mickiewicza 30, 30-059 Krakow, Poland; adglow@agh.edu.pl; 7 Department of Electrical Engineering, Cracow University of Technology, Warszawska 24 Str., 31-155 Cracow, Poland; zbigniew.pilch@pk.edu.pl; 8 Department of Design and Machine Elements, Faculty of Mechanical Engineering, University of Zilina, Univerzitna 1, 010 26 Zilina, Slovakia; frantisek.brumercik@fstroj.uniza.sk

**Keywords:** erosion, wear, corrosion, sand, plug flow, elbow

## Abstract

Erosive wear due to the fact of sand severely affects hydrocarbon production industries and, consequently, various sectors of the mineral processing industry. In this study, the effect of the elbow geometrical configuration on the erosive wear of carbon steel for silt–water–air flow conditions were investigated using material loss analysis, surface roughness analysis, and microscopic imaging technique. Experiments were performed under the plug flow conditions in a closed flow loop at standard atmospheric pressure. Water and air plug flow and the disperse phase was silt (silica sand) with a 2.5 wt % concentration, and a silt grain size of 70 µm was used for performing the tests. The experimental analysis showed that silt impact increases material disintegration up to 1.8 times with a change in the elbow configuration from 60° to 90° in plug flow conditions. The primary erosive wear mechanisms of the internal elbow surface were sliding, cutting, and pit propagation. The maximum silt particle impaction was located at the outer curvature in the 50° position in 60° elbows and the 80° position in 90° elbows in plug flow. The erosion rate decreased from 10.23 to 5.67 mm/year with a change in the elbow angle from 90° to 60°. Moreover, the microhardness on the Vickers scale increased from 168 to 199 in the 90° elbow and from 168 to 184 in the 60° elbow.

## 1. Introduction

Erosion of pipeline is the cumulative removal of material due to the target surface and impinging dispersed phase interaction. It is a critical and convolute issue in the hydrocarbon and mineral processing industry. Erosion can substantially reduce the service life of pipelines and increase the production cost [1]. The erosion induced in multiphase flow is very complicated and is essentially sustained by flow regimes, impact conditions, disperse phase properties, and flow characteristics [2,3]. Complex disperse phase and carrier fluid interactions take place inside pipelines that affect the erosion-induced damage [4]. Aside from the flow conditions, the parameters that considerably affect pipe erosive wear also include the geometrical configuration of pipelines.

Erosion–corrosion in ductile metals has been investigated by many researchers, who found that it was due to the cutting action of the abrasive particles [5,6]. Erosion-induced damage is very much influenced by dispersed phase properties, particle impact conditions, and properties of the target material [7,8].

Sedrez et al. [9] investigated erosion by liquid–solid flow for elbow configurations. The wear pattern identified that the maximum impaction was found at the outer wall near the end of the elbows. Moreover, their computational fluid dynamics (CFD) study and experimental results showed good agreement.

Recently, Owen et al. [10] designed a test methodology for erosion–corrosion analysis in 3D printed 90° elbows in a representative of field flow conditions; it appeared that high flow disturbance would be generated by the protruded samples, significantly influencing hydrodynamics in the flow through the elbow. This could significantly affect local turbulence inside the elbow pipe.

Vieira et al. [11] reported that for gas–sand flow, the highest erosion was identified at 45°. In addition, the sand size had no significant influence on the wear rate in gas–sand flows. It was found that the 300 micron sand degrades material between 1.9 and 2.5 times higher compared to the 150 micron sand. Wang et al. [12] performed a numerical analysis and found that the maximum wear hot spot was also influenced by the particle size due to the fact that the sedimentation will be enhanced with the increase in erodent size; the peak erosion location will be located adjacent to the elbow exit. Vieira et al. [13] observed that a sand size of 300 μm disintegrated 3.7 times more material compared to 20 μm; similarly, the 300 μm sand created 3.1 times more degradation compared to the 150 μm sand particles. In annular flow conditions, the highest erosion was identified at the axial angle of 45° in the outer curvature of the elbow. The influence of erodent size and flow viscosity on material degradation was investigated in [7,14].

X. Cao et al. [15] studied the effect of superficial carrier phase velocities on the erosive behavior of steel pipe bends in water–sand slug flow. They concluded that with the escalation in superficial velocity, the degradation of the maximum eroded specimen reduces. Zahedi et al. [16] observed that for annular flow conditions, erosive wear was incurred with the highest particle wall impaction at 40–50° at the outer radius. 

Surprisingly, there is a dearth of research on the 90° elbow and 60° elbow configuration related to the study of the erosion mechanism of pipes and, more specifically, for silt particles under plug flow. In the plug flow pattern, the bubbles are smaller in size and drive more slowly in comparison with slug flow. This paper aimed to investigate the erosion mechanism for a 1018 carbon steel 90° elbow pipe and a 60° elbow pipe in water–sand plug flow conditions. In this work, a novel erosion test methodology was designed by using representative curve elbow specimens of 90° and 60° elbows; it appeared that in literature, the tests performed on flat specimens mounted inside the elbow influenced the hydrodynamics and increased turbulence inside the pipe. Because existing methods for evaluating the erosion–corrosion of elbows are inadequate, in this research, new experimental procedures are developed to quantify elbow erosion-induced damage under three phase flow conditions.

In this paper, the paint modeling method, microscopic imaging, mass loss quantifications, and hardness testing were used to evaluate the erosive wear of carbon steel 90° and 60° elbow pipelines in plug flow. Furthermore, the erosive wear mechanism of the impact of water–silt–air plug flow was elucidated. In this study, sand of 70 µm size was primarily used to simulate the field operating conditions, with water and air as carrier phases, including the internal erosion of hydrocarbon production and mineral processing industries in multiphase flow, where erosion is due to the sand production such as conditions encountered in oil and gas fields.

## 2. Experiment Procedure and Test Methods

**Figure 1:**
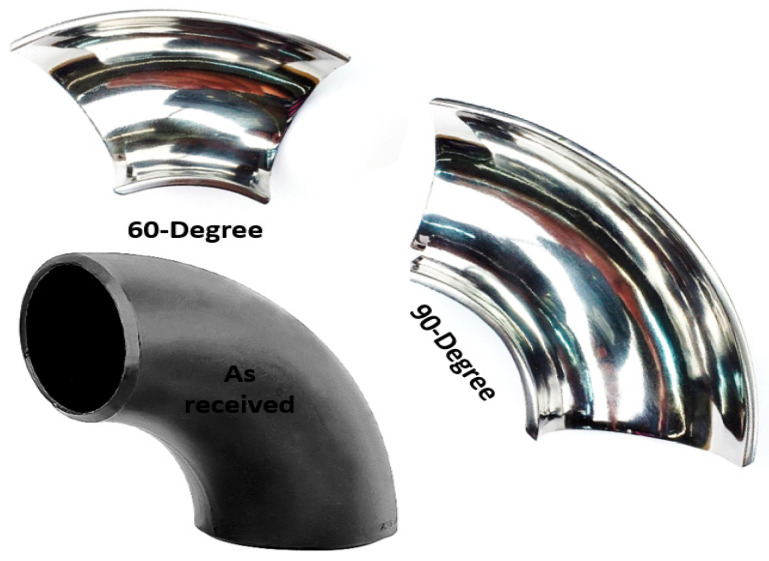
Elbow test specimen.

**Figure 2:**
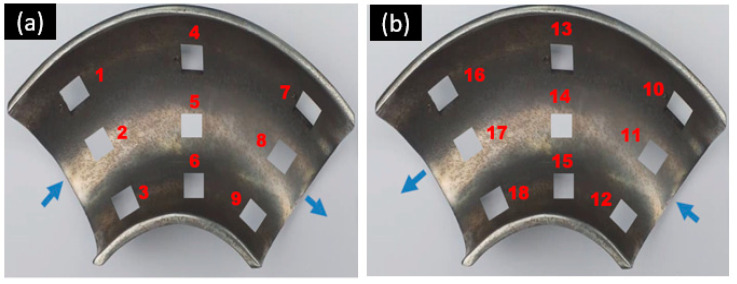
(**a**,**b**) Specimen location after test.

The elbow specimen used in this experimental study was 1018 carbon steel (CS) used with the following composition (in weight %): 0.2% C, 0.26% Si, 0.52% Mn, 0.21% Cr, and 98.12% Fe. The specimens were obtained from the supplier in the form of 90° elbow pipes and 60° elbow pipes. The elbow pipe, specimens in the shape of an axially cut section, as shown in Figure 1, were machined using wire electric discharge machining (WEDM). The finely polished specimens were obtained by grinding and polishing procedures resulting in a low-level surface roughness. The 10 × 10 mm^2^ sizes of the specimens after the test were cut from the different locations of the elbow for microscopic imaging, as shown in Figure 2. A total of 36 specimens were cut from the upper and bottom walls of the 90° elbows and 60° elbows at various locations. The Vickers hardness of the specimens was evaluated under 5 N load using a diamond indenter for a 15 s indentation time using a Leco LM 247AT microhardness tester. The worn surface of the 90° and 60° elbow specimens was studied with a backscattered electron microscope (Phenom ProX, Eindhoven, The Netherlands). Each 10 × 10 mm specimen mass loss was measured using a precision weighing scale to quantify the wear rate after the test. The device used to acquire surface roughness parameters was Mitutoyo SURFTEST SJ-210. Details about the testing procedure were published in our previous work [17,18].

**Figure 3:**
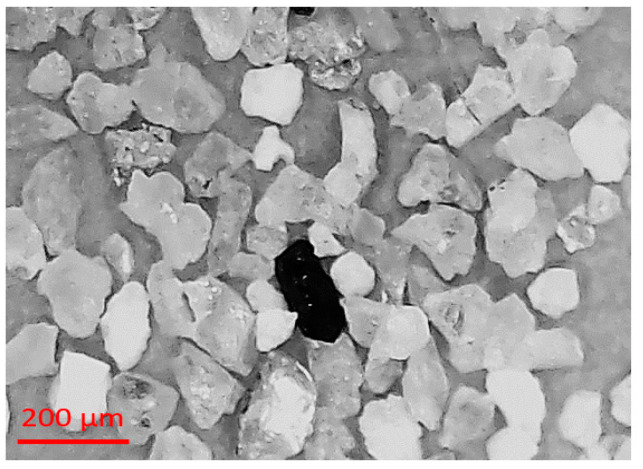
SEM image of silt grain particles.

**Figure 4:**
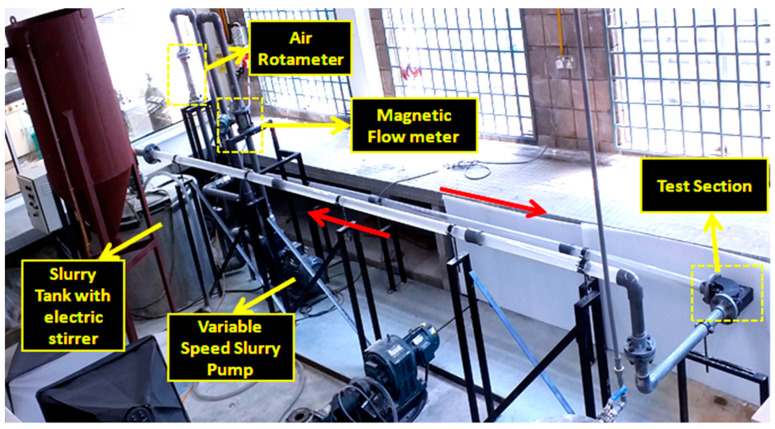
Layout of the experimental setup.

The locally fabricated erosion test flow loop was fabricated in University Technology PETRONAS, Seri Iskandar, Malaysia, using an abrasive pump that used a rubber liner to avoid wear of the pump. The silt particle (silica sand) was used as an erodent for all the experimental evaluations, as shown in Figure 3. The designed flow loop was semi-automated to simulate multiphase flow test conditions. The silt and water carrier phases were mixed in the slurry tank using a stirrer. The dispersed phase and water were then circulated using a variable speed pump in the flow pipelines, as shown in Figure 4. The liquid flow rate was measured by a magnetic flow meter through a 50.8 mm diameter PVC pipe. The test section was designed to mount 90° and 60° elbows for both multilayer paint modeling and erosion investigation for multiphase flow conditions. 

**Figure 5:**
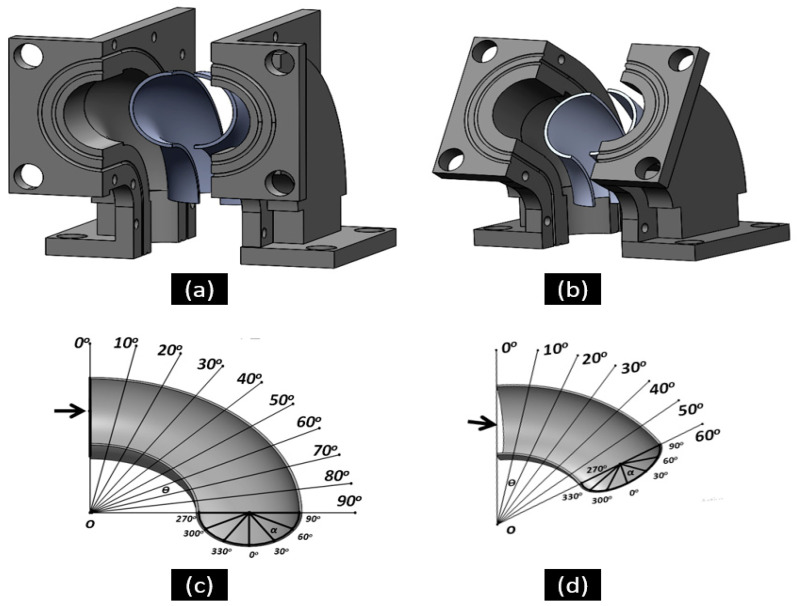
The 90° (**a**) and 60° (**b**) elbow test sections used for the erosion–corrosion studies; (**c**,**d**) definition of the axial angles.

The literature review showed that various erosion test methods have been implemented to study elbow erosion under different flow conditions [19]. Some of the tests use the square sample in which the flat plate is placed at a different axial angle along the elbow pipe. This method leads to huge mass transfer shifts and inaccuracy in measurements and reduces the accuracy of measured data. To resolve this issue, the finely polished elbow sample cut axially into two sections was integrated with the specimen holder in this study, as shown in Figure 5. To quantify the localized erosion rate, the 36 specimens (10 × 10 mm^2^) were cut from different locations on the 90° and 60° elbows, and a standard mass loss test was adopted for erosion rate measurement after the test. The initial mass of all the samples was measured before the test using separate specimens. The location numbers of the specimens are shown in Figure 2a,b. The test section designed in this study used the representative elbow configuration, which provided a better understanding of the erosion mechanism. 

## 3. Results and Discussion

### 3.1. Qualitative Paint Erosion Test

Paint erosion studies were executed on 90° and 60° elbows with a 70 μm particle size for a 90 min flow time. In the paint removal experiment, the inside area of the axially cut elbow specimen was coated with red colored enamel paint and silver colored acrylic paint applied by a spray gun. A digital paint thickness gauge was used to ensure the uniformity of each paint layer. Prior to the paint erosion test, it was necessary to make sure that the paint removal was not caused by flow conditions, and it must be ensured that it was exclusively due to the particle impaction. For tests under nonerosion conditions, it has been concluded that nonerosion flow conditions do not contribute to painting removal in the elbow specimens. Therefore, it can be deduced that the considered paint removal method qualitatively measures the particle impaction regions. Each paint removal test was performed three times to ensure accuracy; it was noticed that the paint erosion pattern tended to be similar for all tests.

**Figure 6:**
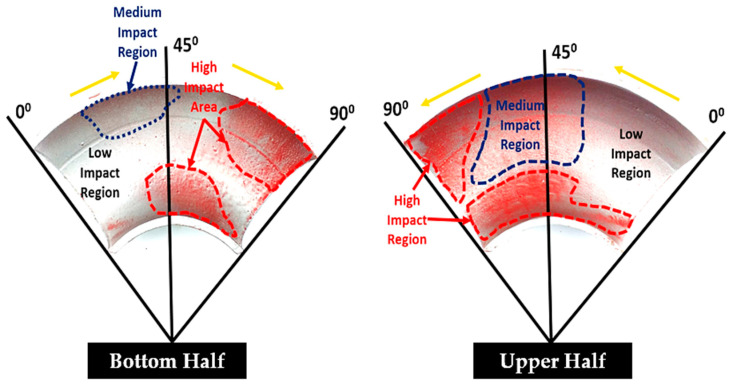
Erosion pattern on the 90° elbow coated with a two-layer paint.

**Figure 7:**
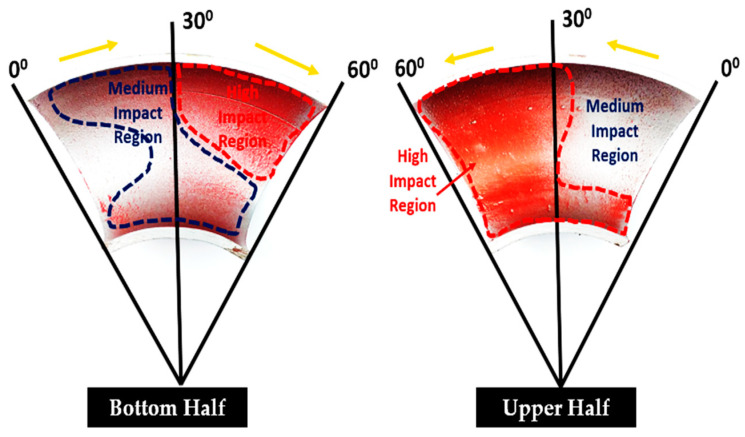
Erosion pattern on the 60° elbow coated with a two-layer paint.

The location of particle impaction in the pipe wall was evaluated after visualization of the paint-eroded regions. Figure 6 shows the paint removal patch in the upper and bottom 90° elbow sections with a 1.5 m/s (liquid velocity) and 0.7 m/s (air velocity) using silt of 70 μm. The high impaction region, on the 90° elbows, tended to be at a location approximately 45° and 90° in the bottom wall and the middle of 0° and 90° in the upper wall of the elbow, respectively. The paint removal marks were nonsymmetric in the top and bottom of the 60° and 90° elbows and the reason air with abrasive particles moving in the upper part and liquid was moving in the bottom section; thus, less erosion occurred in the bottom compared to the top. Figure 7 illustrates the paint erosion pattern in the 60° elbows with silt impact in the bottom and upper half sections for plug flow. For the 60° elbow, the paint was removed between 30° and 60° in the bottom wall and middle of 0° to 60° in the upper section, with greater paint removal pattern clearly seen towards the downstream section in all evaluated cases. In the plug flow regime, the plug body was the key source of erosive wear, because the maximum sand particles were transported by the continuous phase, i.e., water and the plug body had the highest water phase holdup. The highly turbulent plug front with abrasive particles at the elbow curvature can accelerate erosion-induced damage in the top part of pipelines which was evident in the experimental data collected in this study.

### 3.2. Roughness Measurements and SEM Microscopic Imaging

In erosion, the degradation mechanism usually varied from upstream to downstream on the elbow’s internal surface. Identifying the erosion mechanism is important, because it provides a pattern of the degree of wear at different locations. Therefore, after the test, samples were subjected to surface roughness evaluation and microscopic imaging to identify the wear mechanism due to the multiphase flow.

**Figure 8:**
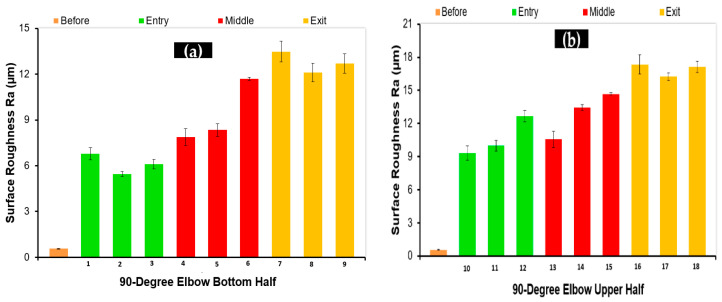
Arithmetic surface roughness values (Ra) before and after the test in 90° elbows: (**a**) bottom; (**b**) top.

**Figure 9:**
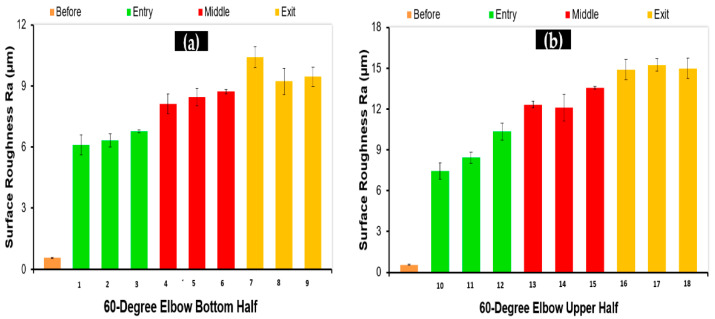
Arithmetic surface roughness values (Ra) before and after tests in the 60° elbows: (**a**) bottom; (**b**) top.

Surface roughness (Ra) values were measured in the flow direction for all 36 specimens cut from 90° and 60° elbows. Three measurements were conducted along the length of the surface of 10 mm of the cut specimen. In Figure 8a, the arithmetic surface roughness values (Ra) of the 1018 carbon steel depending on the location of the bottom elbow section are given. In Figure 8a,b, the arithmetic surface roughness values of the carbon steel varied dramatically as the flow approached from upstream to downstream. The maximum Ra value was observed between 45° and 90° axial angles at the upper and bottom half of the 90° elbows. Hence, it was concluded that carbon steel 90° elbow showed maximum erosion behavior near the outlet, as mentioned in the literature [17]. On the other hand, the Ra of the samples was maximum in the outer wall in both the top and bottom of the elbow pipe. In Figure 9a,b, arithmetic surface roughness values of the 60° elbow samples at the different locations inside the elbow are given. It can be seen that the surface roughness was significantly changed depending on the location. It was clearly observed that surface roughness increased in downstream locations. On the other hand, the maximum surface roughness was between the axial angle of 30° and 60°; however, it decreased in the upstream location. As a result, it can be said that the arithmetic roughness value of the carbon steel generally increased with increases in particle impingement. It was reported in the literature that erosion increases the surface roughness of metallic materials [20].

**Figure 10:**
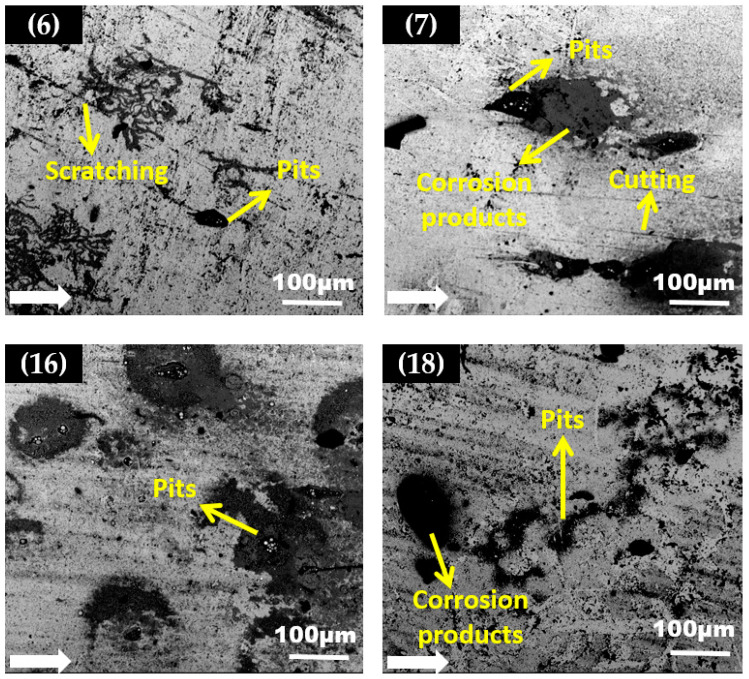
The backscattered electron (BSE) images of a carbon steel 90° elbow after the test.

**Figure 11:**
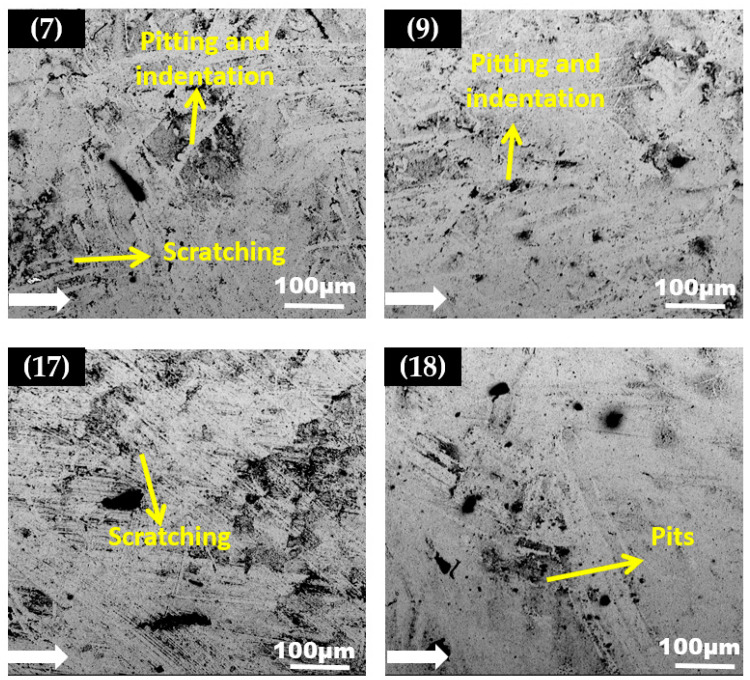
The backscattered electron (BSE) images of a carbon steel 60° elbow after the test.

SEM micrographs of the carbon steel elbow specimen from the upper half and bottom half having maximum arithmetic surface roughness values (Ra) after erosion are shown in Figure 10 and Figure 11. The SEM images showed the surface morphology after the carbon steel elbow was exposed to silt particles under plug flow conditions (a total of 10 h of erosion). Magnified images of sections 6, 7, 16, and 18 with maximum Ra values is shown in Figure 10. Upon impact with silt particles, it locally damaged the surface by the cutting and ploughing action at downstream sections during the erosion and formed pits with corrosion attack at outlet due to the high particle wall impactions.

At the top of the 90° elbow, more areas of pitting and cutting were observed, and this was due to the silt particles impacting with higher kinetic energy which disintegrated materials. Additionally, indentation was also seen at the outlet due to the plastic deformation. Such plastic deformation is usually due to the silt particles redirecting at a curvature and impacting the outer curvature of the elbow pipe. Scratching, pitting, and ploughing are the predominant erosion mechanisms in the downstream section of the elbow. In the outlet, more perforation sites with corrosion attacks were observed which suggests that erosion-corrosion pitting is the dominant erosion mechanism here. Figure 11 presents the erosion mechanism on a carbon steel sample observed after 10 h of testing with silt particles for 60° elbow. After the test, the downstream sections 9 and 18 have smooth areas on the target surface, but in sections 7 and 17 multiple particle impact was visible. In 90° elbow, more pits were detected downstream, pitting corrosion mainly most often at sensitive sites, such as at high particle-wall impaction zones. If these zones are predominant, pitting corrosion is confined to new locations on the surface. Therefore, the number of perforation pits sites will gradually coalesce with the stable pits. In comparison the 60° elbow SEM micrographs showed minimal particle impaction, resulting in the sliding and indentation with relatively small pits. 

Significant disparities in the surface morphologies and development of the pattern of corrosion products at the elbow exit surface after 10 h of tests with silt particles were visible from the SEM microscopy analysis. It can be seen from micrographs that after exposure to the silt particles in the plug flow, the pitting corrosion profile was attributed to a large corrosion zone around the pits, which was covered by a corrosion products layer vicinity of elliptical pits and wide–narrow pits that were evident in the micrographs of the elbow exit section. Moreover, the pits in the 60° elbow grew individually, and the 90° elbows showed sensitive sites that grew up to elongated pits as identified in Figure 10.

The corrosion product concentrated around the pits could be seen in the SEM images. An intriguing observation was that corrosion was detected at the pits because the corrosion product washed out the onset of pits with the increase in particle impact. Notwithstanding, the extent of erosion-induced damage to the 90° elbow was more than those of the 60° elbow surface in the same flow conditions. In the 60° elbow, the erosive wear was less, because the change in the flow direction of the 60° (small angle) elbow was not as abrupt as for the 90° (wide angle) elbow. Apparently, fewer particles are prone to impact the 60° elbow’s outer curvature compared to a 90° elbow outer wall. At the 60° elbow, the flow was redirected more smoothly, which causes the abrasive particles to follow the flow and impact the bottom part with less frequency as compared to the upper part of the elbow.

**Figure 12:**
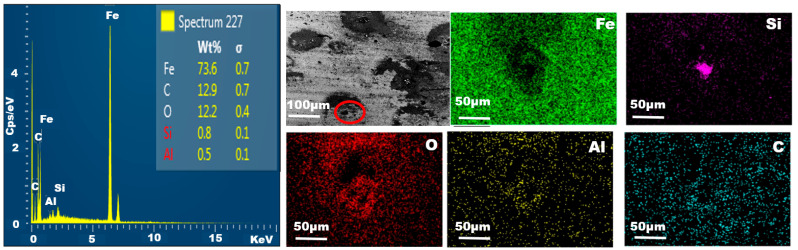
EDS spectra and elemental mapping after erosion in 90° elbow.

The EDS method is used for identification and quantifying elemental compositions after the test for sample #16 in the 90° elbow. The analytical identification of the elements (elemental composition) after the erosion imparted the presence of iron (Fe) and oxygen (O) atoms on sample #16 at the 90° elbows, as shown in Figure 12. In addition, the identification of Si on the eroded surface confirmed that the silt was embedded in the surface after the erosion.

### 3.3. Mass Loss

**Figure 13:**
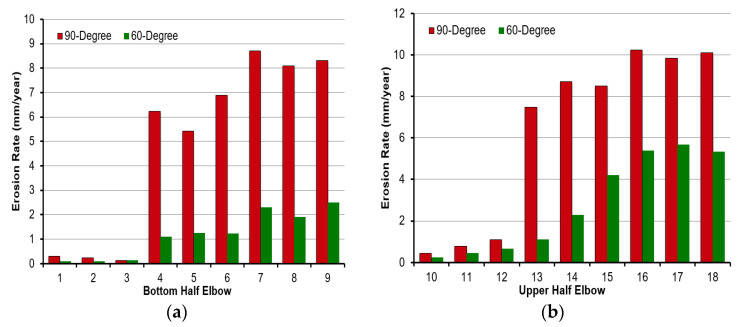
Mass loss in carbon steel elbow section after test: (**a**) bottom; (**b**) top.

Figure 13a,b show the erosion rate for 90° and 60° elbow pipes under plug flow with silt particles. For both elbows, the maximum erosion rate occurred downstream near the outlet. In the 90° elbow, the corresponding maximum erosion rates were 10.23 mm/year compared to the 60° elbow which was 5.67 mm/year. Regardless of the elbow angle, Figure 13b shows that specimens at locations 16 and 18 of the 90° elbow’s upper half provided the highest erosion, and specimens at locations 16 and 17 of the 60° elbow’s upper half reflected the maximum wear rates compared with other positions. This identified that the location adjacent to an outlet for both the 90° and 60° elbows was likely to be eroded during the silt particle’s impact under plug flow conditions. Moreover, the wear rate of the upper half elbow section was more than that of the bottom half. It can be concluded that in plug flow, the top of the elbow downstream is more prone to erosion due to the multiple particle impactions than in other positions. There was an approximately 1.8 times increase in the erosion rate of the maximum impaction region in the 90° elbow compared to the 60° elbow observed for carbon steel for identical flow conditions. The severe silt particle impaction was located at the outer curvature in the 50° position in 60° elbows and the 80° position in 90° elbows in plug flow. 

### 3.4. Hardness Measurements

**Figure 14:**
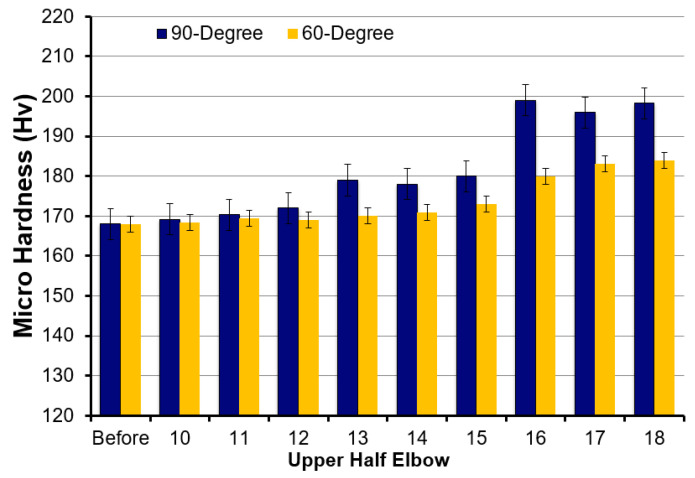
Microhardness of carbon steel elbows’ upper half sections before and after the test.

Figure 14 shows the results of microhardness evaluation at the different locations of the 90° and 60° elbows. As indicated in Figure 14, the hardness of the polished specimen after the test of carbon steel 1018 samples increased due to the impact of silt particles as flow approached downstream. Figure 14 shows a similar trend in the results for both the 90° elbow and the 60° elbow. It was clear from the results that the maximum hardness in both 90° and 60° elbows was, however, observed adjacent to the outlet, which was due to maximum particle impaction. The erosive wear leads to strain that hardened the target surface and the hardness of the carbon steel improved from 168 to 199 in the 90° elbow exit section and to 181 in the 60° elbow exit section on the Vickers hardness scale. The escalation in the hardness value after erosive wear in Figure 14 was accordant with the findings in a previous study [20].

## 4. Conclusions

This paper investigated the influence of elbow angle on the erosive behavior of carbon steel due to the impaction of silt particles in plug flow conditions. A total of 36 specimens were cut from the upper and bottom halves of the 90° elbow and the 60° elbow at various locations. Moreover, a paint removal method, mass loss analysis, microscopic imaging, surface roughness evaluation, and microhardness analysis were employed to study the relationship between the erosion distribution and the elbow angle. The following conclusions were drawn:

1. In plug flow, the erosive wear increased significantly with a change in elbow angle from 60° to 90°. Compared with the 60° elbow, there was an approximately 1.8 times increase in maximum erosion rate in 90° elbows for identical flow conditions;
2. At the top of the 90° and 60° elbows adjacent to the outlet, the erosion maximized due to the redirected flow, and the maximum silt particle impaction was identified at the outer curvature in the 50° position in the 60° elbow and the 80° position in the 90° elbow in plug flow. In the 60° elbow, the erosive wear was less because the change in the flow direction of the 60° (small angle) elbow was not as abrupt as for the 90° (wide angle) elbow;
3. The arithmetic mean surfaces roughness of the samples was dramatically influenced by elbow angle. The surface roughness values and microhardness obtained showed that the surface roughness and hardness of the samples were increased on the top of the elbow compared to the bottom part at the elbow exit. The silt particle impact on the surface of the 60° and 90° elbows in the top part and the subsequent surface damage through scratching, pitting, and material removal resulted in subsequent strain hardening of the surface, which resulted in increased surface roughness and hardness;
4. The microscopic study of the eroded sample showed that the primary causes of wear in the 90° elbow included pitting, ploughing, and cuttings. The microscopic images of the test specimens manifested that pitting, scratching, and indentation eventuated, which is an indication of plastic deformation due to the impact of the silt particles. The progressive effect of pitting, scratching, and indentation increased erosion in the exit of the 90° elbow pipe.

## Data Availability

The data presented in this study are available upon request from the corresponding author.
